# Performance of a sample of patients with Mild Cognitive Impairment (MCI), Alzheimer's Disease (AD) and healthy elderly on a lexical decision test (LDT) as a measure of pre-morbid intelligence

**DOI:** 10.1590/1980-57642015DN93000009

**Published:** 2015

**Authors:** Valéria Trunkl Serrao, Sônia Maria Dozzi Brucki, Kenia Repiso Campanholo, Letícia Lessa Mansur, Ricardo Nitrini, Eliane Correa Miotto

**Affiliations:** 1 Department of Neurology, University of São Paulo, SP, Brazil.; 2 Division of Psychology, Hospital das Clinicas, University of São Paulo, SP, Brazil.

**Keywords:** pre-morbid IQ, language, Alzheimer's disease

## Abstract

**Objective:**

The objective of this study was to describe the performance of healthy elderly patients with aging-related pathologies (MCI) and patients with AD on a lexical decision test.

**Methods:**

The study included 38 healthy elderly subjects, 61 MCI and 26 AD patients from the Neurology Department of the Hospital das Clinicas, Behavioral and Cognitive Neurology Group. The neuropsychological instruments included the episodic memory test (RAVLT), subtests from the WAIS-III (Matrix Reasoning and Vocabulary) to determine estimated IQ, the Boston naming test (BNT) and Lexical Decision Test (LDT).

**Results:**

All groups differed on the MMSE, as expected according to their pathologies, memory tests, naming and estimated IQ. For the vocabulary and the LDT – measures of crystalized intelligence no differences were found.

**Conclusion:**

The LDT demonstrated that lexical decision can be used as a measure of pre-morbid IQ among the individuals assessed in a Brazilian sample.

## Abstract (pt)

**Objetivo:**

O objetivo deste artigo é descrever o desempenho de idosos saudáveis, idosos com patologias relacionadas ao envelhecimento (CCL) e de idosos com DA, em teste de decisão lexical.

**Métodos:**

Participaram 38 idosos saudáveis, 61 CCL e 26 DA, atendidos no Setor de Neurologia do Hospital das Clínicas (GNCC) e do Centro de Referência em Distúrbios Cognitivos (CEREDIC). Os instrumentos neuropsicológicos incluíram teste de memória episódica (RAVLT), Subtestes do WAIS-III (Vocabulário e Raciocínio Matricial) para estabelecer QI estimado, teste de nomeação (BNT) e o Teste de Decisão Lexical (TDL).

**Resultados:**

Todos os grupos diferiram quanto ao MEEM, de acordo com o esperado para as patologias, para os testes de memória, para a nomeação e o QI estimado. No caso do vocabulário e do TDL – medidas de inteligência cristalizada não apresentaram diferença.

**Conclusão:**

O TDL permitiu que se mostrasse que a decisão lexical pode ser uma medida de QI pré-mórbido nos indivíduos avaliados em uma amostra da população brasileira.

## INTRODUCTION

The rapid population aging and resultant rise in the number of elderly has led to a more pressing need for rigorous assessment of cognitive function in this population. In this context, assessing pre-morbid IQ is essential to determine previous ability and thus the degree of current cognitive decline^1^ of this population.

Measuring cognitive impairment is challenging due to variations in cognitive abilities. Performance scores can mask or augment cognitive deficits of individuals with high or low intellectual ability.^2,3^ Consequently, the standards for normative comparisons are limited for detecting impairment and complemented using individual comparisons in assessments of acquired deficits.^4,5^ Ideally these standards should be determined prior to any brain damage but this is practically unfeasible. Health professionals must therefore resort to alternative methods for estimating pre-morbid abilities.^1,2,4,6^

Standardized measures of pre-morbid IQ have proven more reliable and accurate for detecting previous level of functioning.^2^ However, there is an urgent need for improved methods of assessing pre-morbid functioning, adapted to the specificities of different cultures.^7^ The available methods are described in a review by Franzen, Burguess and Smith-Seemiller (1997),^8^ which entail irregular word reading, the lexical decision task and demographic estimates based on regression analysis.^7,9,10^

A widely used instrument is the NART – National Adult Reading Test,^11^ involving verbal reading of 50 irregular words of increasing level of difficulty, for which pronunciation accuracy is assessed. The underlying premise of the NART is that correct pronunciation of words depends on familiarity with them.^2,6,12^ In parallel, Baddeley, Emslie and Nimmo-Smith (1993),^6^ developed the Spot-the-Word (STW) test, consisting of 60 pairs of regular words, one real and the other a pseudoword, for which individuals must select the real word from each pair. The authors claim that the test, by involving lexical decisions, encompasses several parallel processing paths, including semantic, visual and sound aspects, which access the region of the brain that stores verbal or "crystallized" intelligence that is more resistant to brain deterioration. In addition, this task imposes specific cognitive demands involving different semantic aspects of words, such as lexical recognition, morphology, meaning and sense of familiarity with the word.^11^ Another important feature is that the words making up the test are regular, allowing their use in dyslexic individuals or those with learning difficulties. These words also lend themselves to translation for use in non-English speaking countries.^6^ In Brazil, there is currently a dearth of such instruments for assessing pre-morbid IQ.

The objective of the present study was to describe the performance of a group of healthy elderly, a patient group with Mild Cognitive Impairment (MCI), and another with Alzheimer's Disease (AD), on a lexical decision task based on the STW, as a measure of pre-morbid IQ in a Brazilian sample.

## METHODS

This study was carried out in volunteers from the Behavioral and Cognitive Neurology Unit and the Referral Center for Cognitive Disorders of Clinicas Hospital (CEREDIC). All participants signed the Free and Informed Consent Form. The study was approved by the Research Ethic Committee of the Clinicas Hospital of the School of Medicine - HCFMUSP (report 1332/2010).

The study sample included 125 participants, comprising 38 control subjects, 61 MCI patients, and 26 AD patients, aged 60-88 years with 4-24 years of formal schooling.

The clinical groups included individuals diagnosed with MCI^13-15^ and Alzheimer's disease according to the DSM-IV^16^ and the NINCDS-ADRA.^17^ Patients diagnosed with psychiatric or neurological disorders, or in use of psychotropic drugs, as determined by semi-structured interview, analysis of medical records and assessment by a neurologist from the GNCC group, were excluded.

The group of healthy elderly included individuals that scored above the median cut-off value for schooling and age on the MMSE – Mini-Mental State Examination screening test.^18,19^ Exclusion criteria were:

1) dependence for instrumental activities of daily living (IADLs) as assessed by the Measurement of Functional Activities on Older Adults in the Community (Pfeffer scale)^21^ with score >5, and for basic ADLs on the Katz scale;^22^
2) presence of depressive symptoms on the Geriatric Depression Scale (GDS) with score > 5;
3) use of psychotropic medications;
4) unable to complete the study protocol due to physical and/or sensory disability.

**Procedures.** Sociodemographic information was collected from the participants and their companions, whereas data on clinical conditions and medical history were obtained from performance scores and from the medical team. Having met the inclusion and exclusion criteria, participants performed the following cognitive tests:

[1] *Rey Auditory Verbal Learning* (RAVLT). This is a test of verbal episodic memory. The sum of recalls A1-A^5^ was used to measure immediate recall and A^7^ for delayed recall, while the number of correct items from list A was used to assess recognition;^24^
[2] Vocabulary and Matrix Reasoning for estimating current IQ from the Wechsler Adult Intelligence Scale (WAIS-III);^25^
[3] Language naming using the Boston Naming Test (BNT),^26^ scored by correct answers (spontaneous and after semantic prompt).

Finally, participants were submitted to the lexical decision test (LDT) developed based on guidance from Prof. Dr. Alan Baddeley, author of the STW.^6^ This task was carried out based on the Brazilian sociocultural reality, seeking words in the literature which reflected the frequency (high, medium, low) of their occurrence in Brazilian Portuguese.^27^

Besides following recommendation for word frequency in Portuguese, the pairs of stimuli (real word plus pseudoword) began and ended with the same letter and contained the same number of syllables. Factors influencing the processing of single words in situations of recognition were taken into account.^27^

The word list was given to the participants after brief training and explanation about the task. Performance was assessed based on the number of correct answers selected by the participant in silence.

**Statistical analysis.** Sociodemographic information and data obtained from neuropsychological tests were expressed as mean and standard deviation for each group. Cognitive and sociodemographic variables were compared using the Kruskal-Wallis or analysis of variance (ANOVA), dictated by the presence of a normal distribution or otherwise. The gender variable was expressed using absolute and relative frequency while the existence of correlation between gender and the groups was determined using the odds ratio test. Results of neuropsychological tests were compared for the different groups, after controlling for sociodemographic characteristics (age, gender, schooling and socioeconomic level). General linear models were adopted with estimation of factors by least squares, without assuming normal distribution of the neuropsychological tests. For models showing statistical significance, analysis was followed by Bonferroni correction for multiple comparisons to determine which groups differed on the scales. For each group, Spearman's correlation was calculated among results on the neuropsychological tests to determine the relationship between the scales.

Statistical analysis was carried out using the SPSS for Windows (version 20.0) software program. The level of significance adopted was a p-value<0.05.

## RESULTS

The sociodemographic characteristics of the 3 groups in the study are given in Table 1 and show no difference among groups for schooling. The table includes information on cognitive screening and functional activities of daily living. Notably, the 3 groups analyzed had a low rate of depressive symptoms, and only the AD group had scores indicating basic and functional dependence.

**Table 1:** Estimated mean values and standard errors of scales, according to group and results of comparisons.

Variable	Control M (SD)	MCI M (SD)	AD M (SD)	P^1^
Age	67.37 (5.89)	68.92 (6.49)	75.27 (5.89)	<0.001*
Schooling (years)	11.89 (5.07)	9.8 (5.38)	9.58 (4.68)	0.077
MMSE	27.88 (0.62)	26.03 (0.44)	24.34 (0.57)	<0.001*
GDS15	1.83 (0.46)	2.28 (0.33)	2.94 (0.42)	0.211
PFAQ	0.34 (0.79)	1.18 (0.57)	8.41 (0.72)	<0.001*
Katz	6 (0.09)	6.02 (0.07)	5.66 (0.08)	0.003*

* Statistical Significance p<0.05.

1 ANOVA; MMSE: Mini-Mental State Exam. GDS15: Yesavage Scale short form. PFAQ: Pfeffer Functional Activities Questionnaire; MCI: Mild Cognitive Impairment; AD: Alzheimer’s disease.

The results obtained on the RAVLT test by Kruskall-Wallis showed significant difference among the three groups, particularly during immediate recall (A1 to A5) and delayed recall (A 7, see Table 2).

**Table 2:** Estimated mean values and standard errors of neuropsychological scales, adjusted for sociodemographic characteristics and result of comparisons, by group.

Variable	Control M (SD)	MCI M (SD)	AD M (SD)	P^1^	P*		P*		P*
					Control × MCI		Control × AD		MCI × AD
RAVLT – Immediate recall (A1 to A5)	46.55 (2.11)	38.35 (1.52)	25.45 (1.94)	**<0.001**	**0.006**		**<0.001**		**<0.001**
Delayed Recall (A7)	9.53 (0.68)	6.79 (0.49)	2.69 (0.62)	**<0.001**	**0.004**		**<0.001**		**<0.001**
REC	14.56 (0.54)	13.04 (0.39)	10.48 (0.52)	**<0.001**	**0.067**		**<0.001**		**<0.001**
BNT	52.84 (1.54)	49.55 (1.1)	46.44 (1.41)	**0.012**	0.237		**0.009**		0.234

Statistical Significance p<0.05. RAVLT – (A1 to A5): sum of trials on the Rey Auditory Verbal Learning Test. REC: Recognition; BNT: Boston Naming Test.

1 General linear models;

* Bonferroni’s multiple comparisons.

Performance on tests measuring crystalized intelligence, such as vocabulary and the LDT, did not differ significantly on group comparisons (Table 3). However, a significant difference was detected in matrix reasoning and general intelligence quotient (IQ). Post-hoc analysis revealed significant differences on comparisons between the MCI and control group.

**Table 3:** Description of neuropsychological tests and IQ with estimated mean values and standard errors and LDT, adjusted for sociodemographic characteristics and result of comparisons, by group.

Variable	Control M (SD)	MCI M (SD)	AD M (SD)	P^1^	P*		P*		P*
					Control × MCI		Control × AD		MCI × AD
Vocabulary	43.09 (2.4)	38.59 (1.72)	41.96 (2.2)	0.230	0.351		>0.999		0.637
Matrix reasoning	13.12 (0.99)	8.93 (0.71)	9.83 (0.91)	**0.003**	**0.002**		0.054		>0.999
IQ	108.9 (2.46)	99.82 (1.77)	102.89 (2.26)	**0.012**	**0.009**		0.239		0.831
LDT	52.48 (1.2)	49.84 (0.86)	48.96 (1.1)	0.091	0.200		0.095		>0.999
% correct on LDT	87.46 (1.99)	83.07 (1.43)	81.58 (1.83)	0.089	0.200		0.092		>0.999

Data expressed as mean (standard error) adjusted for the variables gender, schooling, age and socioeconomic level. IQ: Intelligence Quotient. LDT: Lexical Decision Test. P1: general linear models.

p*: Bonferroni’s multiple comparisons.

The results showed no significant difference among the groups, confirming the applicability of the LDT for measuring pre-morbid IQ, although further studies are warranted.

Finally, Table 4 shows that both schooling and LDT exerted a statistically significant effect on the result of the current estimated IQ. In conjunction, schooling and LDT explained 66.3% of the variability of the estimated IQ of the control group.

**Table 4:** Estimated IQ in control group by multiple linear regression.

Factor	Coefficient	Standard error	t-Value	P	R ^2^
Constant	–17.55	23.71	–0.74	0.464	0.663
60-word LDT	2.21	0.50	4.466	**<0.001**	
Schooling (years of study)	0.96	0.34	2.831	**0.008**	

LDT: Lexical Decision Test.

## DISCUSSION

The objective of this study was to report the performance of a sample of healthy subjects, MCI patients and AD patients, on a lexical decision task, which showed the potential of the LDT for use as a measure of pre-morbid IQ.

The main results revealed that cognitive performance was directly correlated with pathological condition and age, where the control group had a lower mean age than the patient groups. These findings corroborate the fact that age may be a significant risk factor in dementias.^28^

A statistically significant difference was detected among the groups on the episodic memory test, identifying this as another factor differentiating healthy aging from pathological aging.^29^

Studies on the language area in AD seek to detect changes in semantic-lexical processing, particularly lexical access.^30,31^ Naming deficits are believed to be linked to problems accessing semantic, lexical-semantic and/or phonologic-lexical systems,^32^ as seen in the present study which showed a statistically significant difference among the groups on the naming test applied.

Given that semantic processing encompasses parallel processing pathways, including semantic pathways (visual and phonologic), in developed countries irregular word reading tests have proven valid in comparisons of current intellectual ability with pre-morbid state.^31^ In the context of Brazilian Portuguese, owing to the predominance of regular words, the use of the lexical decision task based on these words would be more suitable for deriving pre-morbid IQ in individuals with suspected cognitive decline.^10,30,31^ Grounded in these concepts, the lexical decision test was devised containing regular words which proved valid for characterizing pre-morbid IQ of the participants in the present study, whose performance on this measure did not differ significantly.

Lexical decision tasks have previously been used as a measure of pre-morbid IQ in other international studies. In Portugal (2010), the TelPI^33^ (irregular word reading test) instrument was created for deriving pre-morbid IQ based on lexical decision. There is also the SLDT^34^ (Swedish Lexical Decision Test) based on the premise that, in the population at large, knowledge of a word is strongly associated with measures of overall cognitive function.

However, although the novel LTD test has proven innovative for the Brazilian population and promising for use in deriving pre-morbid IQ, lack of resources at the time of selecting words for inclusion in the test limited the final instrument. A further limiting factor was the adoption of a methodology more suited for the English language. Future studies involving different samples of the Brazilian population should be conducted to confirm or build on the present results.

Thus, the results of this study showed that pre-morbid IQ was correlated with performance on the LDT which, together with schooling, explained 66.3% of variability in the sample assessed.

This study involved patients from the Referral Center for Cognitive Disorders of Clinicas Hospital (CEREDIC) and from the Behavioral and Cognitive Neurology Unit (GNCC).
